# Middlesex Hospital Prizegiving

**Published:** 1924-11

**Authors:** 


### MIDDLESEX HOSPITAL PRIZEGIVING.

In the absence of Prince Arthur of Connaught, Mr. S. G. Asher presided at the opening of the nineteenth winter session of the Middlesex Hospital Medical School, when the scholarships and prizes awarded during the past year were distributed by Lord Birkenhead. A brisk and concise address on "Some Preventive Aspects of Medicine" was given by Dr. Charles Porter, M.O.H. of Marylebone and lecturer on Hygiene at the Medical School. He had, he said, chosen to speak to the new students on this subject because of a feeling that, once they got into the wards and began to traffic with disease, they might come to regard it as inevitable. They might look upon diseases and disease groups as established things, and so continue to accept them throughout their professional life. Dr. Porter spoke of the desirability of everyone undergoing medical examination at regular intervals. He felt sure that the medical profession would come to realise the value of this, and he advised the turning of attention to the study of the healthy, and of the maintenance and treatment of health.

### Great Traditions.

Mr. Webb-Johnson, the Dean, had another year of progress to record. Various improvements and extensions were in contemplation, and several generous gifts had been received, notably one of £20,000 from Mr. S. A. Courtauld, to endow the University Professorship of Anatomy, and high honours had been obtained in examinations by medical students. Having distributed the prizes, Lord Birkenhead spoke of his pleasure at being present. He had been greatly interested in the statement that all men should be examined seventy-five times up to the age of sixty. He had not reached the age of sixty, and could only recall two occasions on which he had been overhauled. He had made a rough calculation, and he thought that in the course of the next twelve months he should, in order to bring himself into the professor's good books, have to be examined fifty-four times. It was a matter of astonishment to him to realise that the medical school of the hospital had been carrying on its enlightened and beneficent work for 150 years. How much suffering must have been alleviated, anticipated, and prevented in that period needed no imagination, however uninstructed, to understand. During that long period the hospital had maintained its great traditions and *esprit de corps*, which redounded so much to the advantage of those who were privileged to attend the medical school as students. Hospitals rendered a far greater service than the mere attending to the sick, for they contributed to the general well-being of the community by the scientific study of medicine.

### The Birmingham Cancer Campaign.

The Public Health Committee of the Birmingham City Council has decided to distribute to all women in the city a pamphlet, issued by the Cancer Sub-committee, entitled "Important notice to women." The pamphlet, which deals mainly with cancer, treats of premonitory symptoms, and emphasises the importance, if a cure is to be effected, of immediately seeking qualified medical advice.

